# Online health community for change: Analysis of self-disclosure and social networks of users with depression

**DOI:** 10.3389/fpsyg.2023.1092884

**Published:** 2023-03-28

**Authors:** Jiayi Shi, Zhaowei Khoo

**Affiliations:** ^1^School of Foreign Studies, Xi’an Jiaotong University, Xi’an, Shaanxi, China; ^2^School of Mathematical and Computer Sciences, Heriot-Watt University, Putrajaya, Malaysia

**Keywords:** online social support, self-disclosure, social network analysis, content analysis, depression, Weibo

## Abstract

**Background:**

A key research question with theoretical and practical implications is to investigate the various conditions by which social network sites (SNS) may either enhance or interfere with mental well-being, given the omnipresence of SNS and their dual effects on well-being.

**Method/process:**

We study SNS’ effects on well-being by accounting for users’ personal (i.e., self-disclosure) and situational (i.e., social networks) attributes, using a mixed design of content analysis and social network analysis.

**Result/conclusion:**

We compare users’ within-person changes in self-disclosure and social networks in two phases (over half a year), drawing on Weibo Depression SuperTalk, an online community for depression, and find: ① Several network attributes strengthen social support, including network connectivity, global efficiency, degree centralization, hubs of communities, and reciprocal interactions. ② Users’ self-disclosure attributes reflect positive changes in mental well-being and increased attachment to the community. ③ Correlations exist between users’ topological and self-disclosure attributes. ④ A Poisson regression model extracts self-disclosure attributes that may affect users’ received social support, including the writing length, number of active days, informal words, adverbs, negative emotion words, biological process words, and first-person singular forms.

**Innovation:**

We combine social network analysis with content analysis, highlighting the need to understand SNS’ effects on well-being by accounting for users’ self-disclosure (content) and communication partners (social networks).

**Implication/contribution:**

Authentic user data helps to avoid recall bias commonly found in self-reported data. A longitudinal within-person analysis of SNS’ effects on well-being is helpful for policymakers in public health intervention, community managers for group organizations, and users in online community engagement.

## 1. Introduction

Debate continues about the effects of social network sites (SNS) on well-being. An overwhelming amount of literature has focused on the negative consequences of SNS, including (a) intensive use of SNS, which brings experiences of time displacement and overload (van der Schuur et al., 2019; Prestin and Nabi, 2020; Wang et al., 2021); (b) problematic use of SNS, which increases levels of psychological distress, work exhaustion, and suicidal ideation and suicide attempts (McConnell et al., 2018; Froehlich et al., 2020); and (c) upward comparison and envy, led by the tendency to compare with the perfected content of others (Batenburg and Das, 2015; Spitzer et al., 2022). On the other hand, accumulating research has highlighted SNS’ positive effects, including (a) online social support, which serves as a coping mechanism against negative thoughts and events (Oh et al., 2014; Bi et al., 2020); (b) reduced riskiness of self-disclosure, facilitated by SNS’ publicity, broad reach, usability, and immediacy (Pan et al., 2017; Li et al., 2019; Chu et al., 2022); and (c) inspiration and enjoyment, when users reach out for social relationship maintenance (Kim and Kim, 2020; Myrick and Willoughby, 2021).

Given the omnipresence of SNS and their dual effects on well-being, a key research question with practical and theoretical importance is to investigate the various conditions by which SNS may either enhance or interfere with well-being (Ramamoorthy et al., 2021; Valkenburg et al., 2021; Hall and Liu, 2022; Parry et al., 2022). It is to understand why, how, and/or for whom SNS use is associated with well-being (Valkenburg, 2022). As pointed out by some recent reviews, this line of argument has predominantly relied on cross-sectional designs and self-reported measures of SNS use, which can be subject to recall (Hall and Liu, 2022; Parry et al., 2022; Valkenburg, 2022). Similarly, other reviews indicate that studies on SNS use tend to focus mainly on quantity (e.g., time spent, frequency of use) without much consideration of users’ content or communication partners (Yoon et al., 2019; Cingel et al., 2022; Oliver, 2022).

We examine users’ self-disclosure (content) and social networks (communication partners) to fill the gap. We focus on a group of engaged users who have been within an online community for half a year, thus enabling within-person observation to assess SNS’ effects. Users’ authentic data also helps to avoid recall bias commonly found in self-reported data (Cingel et al., 2022; Parry et al., 2022). Longitudinal within-person analyses can facilitate public health intervention as more people turn to SNS for online support (Leung et al., 2014; Yang et al., 2017; van der Schuur et al., 2019). This paper is structured as follows. We first provide the theoretical framework, followed by methods, results, and discussion.

## 2. Theoretical framework

This section will present our theoretical framework. We will demonstrate— between social network analysis and self-disclosure studies—a shared focus on social support embedded in interpersonal relationships, providing the basis for our mixed use of the two approaches to assess SNS’ effects on well-being.

### 2.1. Social support and SNA

With the growing availability of online support communities, questions have been raised about their effects, calling for a more nuanced understanding of online social support. Studies have shown that online users receive emotional comfort, health-related information, belonging support, and health awareness (Oh et al., 2014; Hou et al., 2020; Liu et al., 2021; Lei et al., 2022). Users form supportive, asynchronous, and fulfilling relationships that reduce anxiety and improve life satisfaction in various conditions, such as cancer (Kim et al., 2013; Myrick et al., 2015), depression (Pan et al., 2020; Lu et al., 2021), postpartum depression (Lei et al., 2022), and HIV (Ho et al., 2017; Wang et al., 2018). On the other hand, some have indicated that passive consumption of online support can result in ineffective coping, unwanted help, negative responses, and contradicted suggestions (Batenburg and Das, 2015; Hou et al., 2020; Lin and Li, 2021).

The conflict in the literature lies in the primary focus of understanding social support as an attribute of people surrounding the individual, including the types, amount, and impact of support on the individual (Oh et al., 2014; Zhang and Yang, 2014; Wright, 2016; Liu et al., 2018; Lin and Li, 2021). As a result, these approaches have overlooked social support’s relational and structural aspects. As with offline social support, emerging data have increasingly suggested that the effects of online social support are shaped and contextualized by interpersonal relationships between providers and recipients (Eysenbach et al., 2004; Gibbs et al., 2010; Myneni et al., 2016; Joglekar et al., 2018; Levonian et al., 2021). Developing interpersonal relationships is a prerequisite or cause of network structure, and both can affect feelings of social support (Lakey and Orehek, 2011; Ali et al., 2015; Hall and Liu, 2022). Online support contributes to well-being when users engage in ways that foster meaningful interpersonal connections (Reifegerste et al., 2017; Li et al., 2019; Lin and Li, 2021).

Recent developments in Social Network Analysis (SNA) enable researchers to understand how relational data works. SNA defines social networks as the structural framework within which social support is provided or not based on the ties (edges) between individuals or groups (nodes) (Cobb et al., 2010; Lin et al., 2014; Li and Xu, 2020). SNA describes social relationships in terms of contacts and connections. Relational connections enable the exchange between individuals or groups and their feelings, thoughts, and behaviors (Xu and Zhang, 2016; Lin and Li, 2021; Qu et al., 2022). Throughout these connections, individuals and groups share inherent resources, including social support (Phung et al., 2013; Zhang et al., 2017; Li and Xu, 2020; Pan et al., 2020).

In contrast to its potential with relational data, few SNA studies have looked into users’ content in online communities about diseases or the characteristics of users’ networks (Ramamoorthy et al., 2021). Some of the limited studies have examined social support on social network sites. Some have focused on how individuals are surrounded by relationships that affect their health behaviors, social supports, and health outcomes (Cobb et al., 2010; Yang et al., 2017; Froehlich et al., 2020; Li and Xu, 2020; Liu and Yeo, 2021). Some have examined how network structures can facilitate social support, disseminate health-related information, and increase health awareness (Joglekar et al., 2018; McConnell et al., 2018; Bi et al., 2020; Lei et al., 2022).

Publications that concentrate on SNA of online social support more frequently use static projections of networks, in which they use community detection approaches or identify groups at a specific moment in time as the means of describing the networks (Yang et al., 2017; Froehlich et al., 2020). Nevertheless, longitudinal networks are better analyzed through snapshots since networks are temporal objects whose edges and nodes change over time (Leung et al., 2014; Ramamoorthy et al., 2021; Yang et al., 2021). Online support networks develop in phases (early and late), with some users remaining engaged for a long time while others join and leave. In each phase, network-level attributes can explain group communication and support network, including connectivity, global efficiency, average clustering coefficient, and degree centralization (Froehlich et al., 2020; Li and Xu, 2020; Yang et al., 2021); and node-level attributes can evaluate the role of individuals and their dynamic interaction, including degree and betweenness centralities (Cobb et al., 2010; Joglekar et al., 2018; Li and Xu, 2020).

SNA investigates the interpersonal communication network in disseminating information, transmitting social support, and affecting health behavior and outcome. Content produced by users in the network deserves equal attention, including, most importantly, users’ self-disclosure.

### 2.2. Self-disclosure and social support

People nowadays increasingly disclose themselves on social network sites (SNS). The process of revealing the previously unknown to others through user-generated content is called self-disclosure (Gibbs et al., 2010). Literature on self-disclosure focuses on self-disclosure’s content and impact (Huang, 2016; Chu et al., 2022).

Researchers can analyze mental health outcomes that reflect users’ well-being based on self-disclosures on SNS. Several self-disclosure studies focus on detecting depression, a common and important negative indicator measured in social media and well-being literature (Balani and De Choudhury, 2015; Xu and Zhang, 2016; Leis et al., 2019; Lu et al., 2021). In that regard, well-being can be defined as a spectrum on which there is both the presence and absence of mental health. Well-being can be measured by various positive and negative indicators, such as life satisfaction or depression (Houben et al., 2015; Hall and Liu, 2022; Valkenburg, 2022). Psychological studies examine the concept of well-being in the context of specific mental disorders (e.g., depression, panic disorder, post-traumatic stress disorder) that interfere with individuals’ sense of autonomy, self-esteem, and growth (Ryff, 2014; Zhang et al., 2022). A person suffering from depression may experience and express persistent sadness, physical pain, shame, negative emotions such as anger and self-loathing, a loss of interest in daily activities, and suicidal thoughts in certain circumstances (Yoon et al., 2019). Consequently, researchers have found that using first-person singular pronouns, negative emotion words, and death words are important self-disclosure attributes that help reveal depression symptoms (Rosenquist et al., 2010; Leis et al., 2019; Tadesse et al., 2019).

Another line of argument relates to the impact of self-disclosure on well-being (Luo and Hancock, 2020; Chu et al., 2022). By addressing negative emotions, self-disclosure can ease depressive moods and improve mental well-being, making it a valuable therapeutic ingredient. Individuals’ ability to recover from adversity can be enhanced by sharing their stories with others. According to Pennebaker et al. (2015), participants assigned to a writing assignment of traumatic and upsetting experiences showed benefits to their immune systems. Discourse about emotionally loaded traumatic events can be a safe way to confront mental illness.

The literature, however, provides contradictory findings regarding the benefits of self-disclosure on SNS. Online communication’s anonymity, publicity, wide reach, and immediacy make self-disclosure a double-edged sword. On the one hand, researchers have suggested that bloggers benefit from self-disclosure in maintaining and extending their human relations, which improves mental well-being (Lee et al., 2019; Luo and Hancock, 2020). Posting on SNS tends to reduce the perceived riskiness of self-disclosure, thus encouraging people to express themselves openly, vent negative feelings, and seek social support (Huang, 2016; Ho et al., 2017; Malloch and Taylor, 2018; De Simoni et al., 2020). On the other hand, while online communities involve the interaction of at least two subjects, these objects are typically unclear and unstable unless they have been targeted and notified. A long-term absence of objects during self-disclosure may lead to self-isolation and loneliness for some users (Gibbs et al., 2010; Luo and Hancock, 2020; Chu et al., 2022). When users feel ignored or excluded in online interpersonal interaction, they may experience cyberostracism (Zhang et al., 2022). Aside from this, when users disclose personal information (such as feelings, experiences, and thoughts), they expose themselves to cyberbullying risk since other users may have different values and cognitive preferences. Thus, users in online communities need to be able to establish a friendly semantic environment for digital communication in order to avoid negative consequences of self-disclosure, such as cyberbullying and cyberostracism (Gibbs et al., 2010; Pan et al., 2017).

Reconceptualizing self-disclosure in relational contexts may provide a valuable framework for reconciling the conflict in the literature. More recently, some researchers have recognized the critical need to study the mediation effect of social support in the self-disclosure to well-being link, highlighting the fact that self-disclosure and its associations with well-being are complicated and context-dependent (Hou et al., 2020; Chu et al., 2022; Lei et al., 2022). However, additional research on self-disclosure incorporating users’ relational contexts (i.e., communication partners) is urgently needed, especially in light of online communities’ rising popularity. Social deficiencies among offline social circles or limited access to like-minded individuals have made online communities especially attractive for social connection (Liu et al., 2018). For example, compared to face-to-face support networks, online health communities are frequently used by individuals whose primary social network (i.e., friends and family) has limited knowledge of their health condition (Wright, 2016; Yoon et al., 2019; Pechmann et al., 2020). The study of how users’ self-disclosure content relates to social support in relational contexts has important implications for improving social connections within online communities.

Collectively, as we have synthesized in this section, social network analysis and self-disclosure studies outline a critical need to understand SNS and their effects on well-being in a network of interpersonal relations that enhance or interfere with social support. Social support on SNS is perceived, received, and achieved in a network of relationships surrounding the users, whose self-disclosure triggers partner responsiveness and group interaction.

Our research aims to assess SNS’s effects on well-being by accounting for users’ content (self-disclosure) and communication partners (social networks) in the online support community. We use a mixed method of social network analysis and content analysis. Based on our literature review, mixing the two approaches is based on their shared emphasis on social support embedded in interpersonal relationships. In the following sections, relevant literature will be used to explain and support the findings with regard to three research questions. We will utilize SNA to answer research question 1, a content analysis of self-disclosure to answer research question 2, and a comprehensive analysis of users’ networks and self-disclosure to answer research question 3. We will describe our analytical procedures and the results based on the following research questions:

1. How would the dynamics of network structure strengthen or hinder social support?
2. How would the changes in users’ self-disclosure reflect their mental well-being?
3. How can users obtain more social support through self-disclosure?

## 3. Materials and methods

### 3.1. Data collection and methods

For the current study, we choose a social network site called the Depression Supertalk^1^ in Weibo. For a longitudinal comparative analysis, we collect data during July 2021 (phase 1) and January 2022 (phase 2). To have a within-person research design, we group the overlapped users in two phases and refer to them as engaged users (*N* = 221). As suggested by some research, engaged users who stay within one community tend to develop a sense of community, which refers to the degree to which an individual identifies with members within the community (Eysenbach et al., 2004; Carron-Arthur et al., 2015). We use a customized Web crawler (script written in Python) to extract engaged users’ attributes, including user ID, gender, posts, in-replies, out-replies, number of likes, number of retweets, and comment users.

We construct the social network formed by the post-reply connections of the engaged users and others. We name the constructed network the Depression Supertalk Network (DSN), a multi-directed network with self-loops and multiple edges. We compute and compare the DSN in phase 1 and phase 2 to show its dynamics, drawing on a set of well-established metrics in SNA (see section 3.2). We use Anaconda Python 3.6^2^ for data collection, NetworkX 2.2^3^ for network analysis and visualization, and GraphPad Prism 9.3.1^4^ and SPSS 27^5^ for statistical analysis.

We segment and tokenize users’ posts and comments for content analysis with Butter^6^ and feed the data into the Chinese version dictionary of Linguistic Inquiry Word (SC-LIWC),^7^ a validated and well-adopted toolkit for psychometric analysis in mental expression research (Lieberman, 2007; Pennebaker et al., 2015; Xu and Zhang, 2016; Lumontod and Robinson, 2020). LIWC classifies the input words into four categories: linguistic processes (e.g., pronouns, adverbs), psychological processes (e.g., emotions and cognitive process), personal concerns (e.g., biological concerns, death), and spoken (everyday language use). The resulting categories are standardized as the occurrence rate of corresponding categorical words in the messages. Further, these categories can be used as linguistic and psychometric indicators of depression.

### 3.2. Measures

We use a set of well-established network-level and node-level attributes to analyze the dynamics of the DSN (see Table 1). Network-level attributes include global efficiency, average clustering coefficient, and degree centralization; node-level attributes include in−/(out−) degree, in−/(out−) degree centrality, and betweenness centrality.

**Table 1:** Data and variable description.

Variable^A^	Operational definition	Description
*Social network data*		
Post	The number of posts by a user.	The total number of posts contributed to the forum indicates members’ participation in the community.
In-reply	The number of comments left for a user’s post.	It proxies social support received by the user, especially information support.
Out-reply	The number of replies a user gives to others.	It proxies the amount of social support a user provides to others.
Like	The number of posts that are rated favorable by other users.	It gauges the amount of social support received by a user, especially emotional support.
In-degree	The number of inbound links directed toward a node.	It proxies to what extent others contact a user in the network. It gauges the amount of social support received by a user.
Out-degree	The number of connections initiated by a user (outbound links from a node) in his/her social network.	It proxies the social support a user offers to other members.
Betweenness-centrality	The proportion of the shortest paths between pairs of two nodes traversing through a node.	It proxies the importance of a node for the interactions of other nodes in the network.
(Out−/in-) degree centrality	A node’s (out−/in-) degree centrality is the fraction of nodes its edges are connected to.	It gauges the importance of a given node in the information flow.
Global efficiency	It calculates the average geodesic distance, i.e., the mean value of the distance between all pairs of nodes.	Global efficiency is inversely related to the topological distance between nodes and proxies the information transfer efficiency of the network.
Average clustering coefficient	It is the mean of the fraction of ties among a node’s contacts over the possible number of ties between them.	It quantifies the extent to which neighboring nodes are connected.
Degree centralization	Degree centralization is the overall integration or consistency of the graph.	It measures the distribution of positional advantages of nodes in the network.
*Self-disclosure*		
Quantity		
Breadth	1st person singular pronouns (i); death words (death); biological process (bio)^B^	Bio-words refer to primarily factual information related to health information users share, including symptoms, treatments, and embodied experiences of depression. Death words are linked to suicidal ideation and suicide attempts. First-person singular pronouns reflect self-references.
Duration	*Writing length (WC)*	Writing length represents the degree of community involvement. It proxies a desire for social support, for it denotes the intimacy and the emotional attachment the members have toward the online health community.
Frequency	Active day	Count as the number of days the users post in the community. It gauges users’ self-disclosure frequency and attachment to the community.
Quality		
Valence:	Negative emotion (negemo); positive emotion (posemo); cognitive process (cogproc)	Positive and negative valences reflect the hedonic aspect of well-being. Cognitive processing words reveal the depth and complexity of users’ thinking, demonstrating the eudaimonia aspects of well-being.
Authenticity	Adverb (adverb)	Adverbs emphasize the degree and extremeness. Adverbs indicate sentiment and authenticity in self-presentation.
Intention	Informal words (informal)	Informal language use emphasizes in-group membership and a commitment to group integration. It enhances support seekers’ legitimacy in soliciting help.

A For the comparative design of this study, all variables were recomputed in both phases (July 2021 and January 2022).

B LIWC code in square.

To analyze the topological position of the users, we calculate in-degree centrality, out-degree centrality, and betweenness centrality. In the DSN, a unique node in the network represents a unique user ID. An edge between two nodes represents the existence of a reply-to relationship between the two corresponding nodes. A directed arrowhead stands for the direction of the comment: an inbound link represents in-reply to the user, and an outbound link represents the user’s out-reply to others. In-degree counts inbound links directed toward a node, representing the extent others contact a user. It proxies the amount of social support a user receives (Yang et al., 2017; Froehlich et al., 2020). Out-degree is the number of connections initiated by a user, which proxies the social support a user offers to others (Zhang and Yang, 2014; Li and Xu, 2020). A node’s degree centrality is the fraction of nodes its edges are connected to. Betweenness centrality indicates the extent to which a node occupies an intermediary position on the shortest paths connecting other nodes and serves as a potential go-between. Depending on the betweenness score, some nodes act as a bridge for clusters, while others remain isolated within a local cluster (Froehlich et al., 2020; Yang et al., 2021).

For the view of the whole network, we analyze global efficiency, average clustering coefficient, and degree centralization. Global efficiency concerns network closeness. It calculates the average geodesic distance, i.e., the mean value of the distance between all pairs of nodes (Knoke and Yang, 2008). The average clustering coefficient is the mean of the fraction of ties among a node’s contacts over the possible number of ties between them (Bi et al., 2020). Degree centralization is the overall integration or consistency of the graph (Li and Xu, 2020).

In terms of content analysis, we use LIWC as a language model to capture linguistic and psychological attributes in users’ self-disclosure, quantity (duration, frequency, breadth), and quality (valence, authenticity, intention) (see Table 1).

*Duration*: The writing length reflects the users’ self-disclosure through sharing personal thoughts, ideas, and feelings with others. It proxies users’ amount of communication and activeness in involvement (Lieberman, 2007; Batenburg and Das, 2015; Pan et al., 2017). LIWC captures the writing length as word count, i.e., the mean of the number of words in a post.

*Breadth*: Breadth of self-disclosure related to the range of topics covered within self-disclosure. Some words provide experiential information about psychosocial experiences of depression, including first-person singular forms, biological process words, and death words (Rosenquist et al., 2010; Leis et al., 2019; Tadesse et al., 2019). Accordingly, we use the LIWC score on first-person singular forms (i-words), biological process words (bio-words), and death words. Biological process words include sub-dimensions, including body, health/illness, sexuality, and ingesting.

*Frequency*: Active day counts the number of days the user posts in the group. It shows users’ attachment to the community. It proxies users’ desire to self-present and community-involvement (Liang et al., 2019).

*Valence*: Psychological well-being includes psychological adjustment and negative maladjustment (Calancie et al., 2017). It conceptualizes hedonic aspects (e.g., positive and negative feelings) and eudaimonic well-being (e.g., cognitive assessment of life) (Houben et al., 2015; Oliver, 2022). LIWC captures the former as negative and positive emotion words, in which negative valence systems consist of anxiety, anger, and sadness. The latter is captured by cognitive processing words and further classified into causation, insight, discrepancy, inhibition, tentativeness, and certainty.

*Authenticity*: Adverbs (e.g., very, really) often emphasize the degree and extremeness. Adverbs indicate sentiment and authenticity in self-presentation. Absolute words, such as ‘always’ and ‘never,’ are also reliable markers for diagnosing mental illness (Huang et al., 2019).

*Intention*: Informal language emphasizes in-group membership and a commitment to group integration (Pan et al., 2017). It also enhances support seekers’ legitimacy in soliciting help. LIWC captures it as ‘informal language’ with five sub-dimensions: swear words, netspeak, assent, nonfluencies, and fillers.

## 4. Results

To facilitate the presentation and interpretation of the results, we will divide this section into three parts, each focused on a research question.

### 4.1. Results of social network analysis (SNA)

1. How would the dynamics of network structure strengthen or hinder social support?

We analyze engaged users’ longitudinal networks in snapshots: phase 1 (July 2021) and phase 2 (January 2022). In phases 1 and 2, apart from 221 engaged users, there were 815 users and 868 users who interacted with the engaged users in the DSN, respectively.

Table 2 provides a descriptive analysis of the node-level attributes of the 221 engaged users. Engaged users’ in-degree (received support) increased from phase 1 (*M* = 6.30, *SD* = 11.65) to phase 2 (*M* = 7.27, *SD* = 18.30), but this difference did not reach statistical significance, *t*_220_ = −0.886, *p* = 0.38. Conversely, a paired t-test indicated a significant difference between out-degree in phase 1 (*M* = 0.96, *SD* = 0.99) and phase 2 (*M* = 0.64, *SD* = 0.91), *t*_220_ = 3.76, *p* < 0.01. The in-degree range was larger than the out-degree in both phases, and there was more variability across the users’ in-degree than out-degree. The range and variability of in-and out-degree describe whether the population is homogeneous or heterogeneous in structural positions. While the coefficient of variation was higher for in-degree than out-degree in both phases, it shows that the population was more homogeneous concerning the out-degree (offering support to others) than in-degree (receive support from others).

**Table 2:** Descriptive analysis of node-level attributes of the engaged users (*n* = 221), *M* (SD).

Node attributes	Phase	Range	*M* (SD)	Variance	Coefficient of variation
In-degree	1	1–112	6.30 (11.65)	135.68	184.89%
	2	1–255	7.27 (18.30)	334.76	251.66%
Out-degree	1	0–8	0.96 (0.99)	0.99	103.33%
	2	0–6	0.64 (0.91)	0.83	142.34%
Degree	1	1–114	6.22 (12.04)	144.90	193.57%
	2	1–225	7.27 (18.44)	340.06	253.65%
Betweenness centrality	1	0–0.34	0.01 (0.03)	0.001	294.18%
	2	0–0.45	0.01 (0.03)	0.001	376.97%

Table 3 presents a descriptive analysis of the network-level attributes. The DSN developed better connectivity and non-centric group interaction, evidenced by increased global efficiency, higher average clustering coefficient, and low degree centralization. Global efficiency proxies social transmission and is inversely related to the topological distance between nodes. In phase 2, the global efficiency increased from 0.20 to 0.24, indicating a closer topological distance between nodes and better network connectivity. The average clustering coefficient is a measure more weighted to the local environment of each node, as it quantifies the extent to which neighboring nodes connect (Qu et al., 2022). It measures the probability that two friends of a user are also friends. The average clustering coefficient of the DSN increased from 0.0026 to 0.0044, indicating better network connectivity in phase 2. Degree centralization shows the overall integration or consistency of the graph (Li and Xu, 2020). It measures the distribution of positional advantages of nodes. Highly centralized networks feature one or few individuals monopolizing network interactions (e.g., An extreme example resembles a star network, when all individuals connect to only one individual). The DSN featured low degree centralization (phase 1 = 0.11; phase 2 = 0.23), which suggests a low distribution of positional advantages of nodes. In other words, the low degree centralization showed that the DSN featured non-centric group interaction.

**Table 3:** Descriptive analysis of the network-level attributes in two phases.

Network attributes	Phase 1	Phase 2
Edges	1,316	1,551
Nodes	1,036	1,089
Global efficiency	0.20	0.24
Average clustering coefficient	0.0026	0.0044
Degree centralization	0.11	0.23
Communities	58	50

**Figure 1:**
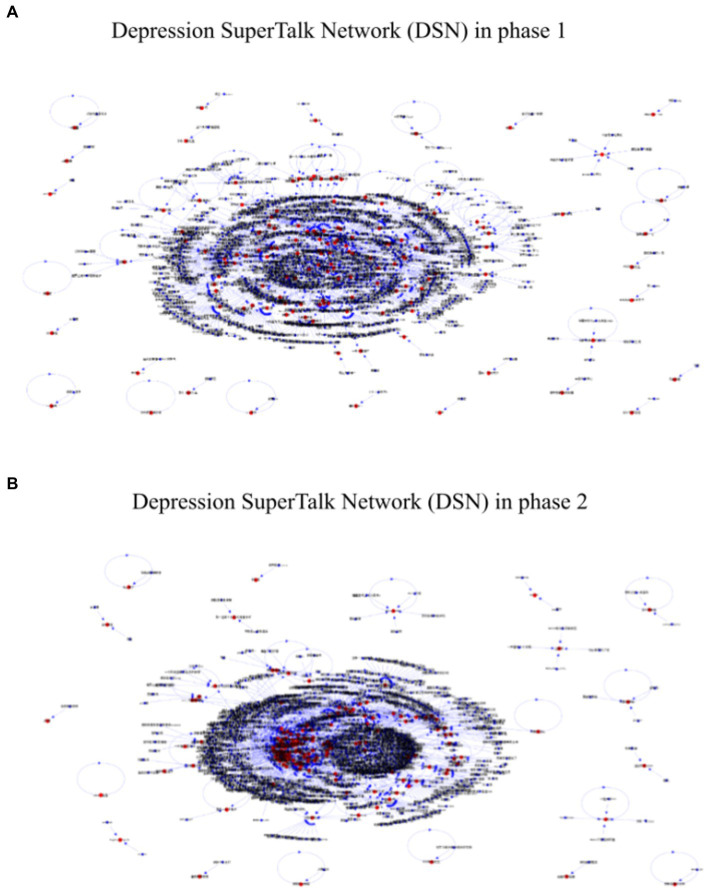
Depression SuperTalk Network (DSN) in **(A)** phase 1 (July 2021) and **(B)** phase 2 (Jan 2022). Red nodes (*n* = 221) indicated engaged users who were in the DSN in both phases, and black nodes (*n* = 815 in phase 1; *n* = 868 in phase 2) indicated users who interacted with engaged users. Edges (*N* = 1,316 in phase 1; *N* = 1,551 in phase 2) between nodes were drawn based on reply connections. A directed arrowhead stands for the direction of the reply, with an inbound link representing in-reply to the user and an outbound link representing out-reply the user gives to another user. Note that red nodes clustered together in phase 2. There were more inbound links than outbound links for red nodes. Also, there were fewer self-loops in phase 2.

To demonstrate the dynamics of the DSN, we compute the social network structures of phase 1 (Figure 1A) and phase 2 (Figure 1B). We differentiate the engaged users as red nodes and users who interacted with the engaged users as black nodes. Users increased slightly, indicating the expansion of the DSN. The clusters of red nodes in phase 2 showed that engaged users developed a denser network. A directed arrowhead stands for the direction of the comment, with an inbound link representing in-reply to the user and an outbound link representing out-reply the user gives to another user. Inbound links (phase 1: *n* = 4,367; phase 2: *n* = 4,860) overwhelmed outbound links (phase 1: *n* = 1700; phase 2: *n* = 1,412) for engaged users, suggesting they received much more social support than they offered support to others. Significant correlation between in−/out-reply attributes showed reciprocal group interaction in the DSN (phase 1: *r*_219_ = 0.69, *p* < 0.001; phase 2: *r*_219_ = 0.68, *p* < 0.001). Also, fewer self-loops in phase 2 meant an overall increase in group interaction.

**Figure 2:**
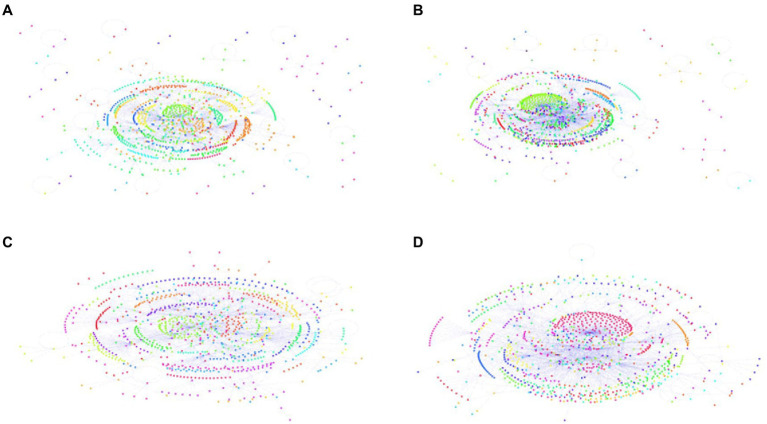
Louvain communities detection of the DSN **(A)** all communities in phase 1, **(B)** allcommunities in phase 2, **(C)** sub-communities with over 10 nodes in phase 1, **(D)** sub-communities withover 10 nodes in phase 2. The nodes in the same community calculated using the Louvain algorithmwere depicted using the same color. Note how communities were denser and appeared darker in phase 2 **(B)** than phase 1 **(A)**. Sub-communities with over 10 nodes were denser and appeared darker in phase 2 **(D)** than phase 1 **(C)**.

We use the Louvain algorithm to identify the communities in the DSN (Blondel et al., 2008). The Louvain algorithm draws on maximizing modularity. A community’s modularity is measured by its density at the inner edges compared to the other edges in the network. Louvain’s algorithm starts with small communities and iteratively merges them into communities with maximum modularity. There were 58 communities in phase 1 (see Figure 2A) and 50 in phase 2 (see Figure 2B). Users clustered in more extensive and denser communities. The DSN increasingly formalized solid communities and strong ties inside the communities. Smaller communities progressed into larger ones through cohesive social ties, interactions, and associations. The density of users within communities was higher in phase 2 (*M* = 21.78, *SD* = 29.72, range 1–189) than in phase 1 (*M* = 17.86, *SD* = 21.08, range 1–98). The density of users with more than 10 nodes was also higher in phase 2 (*M* = 36.29, *SD* = 33.23, range 11–189), compared with that of phase 1 (*M* = 32.40, *SD* = 20.47, range 12–98) (see Figures 2C,D).

**Figure 3:**
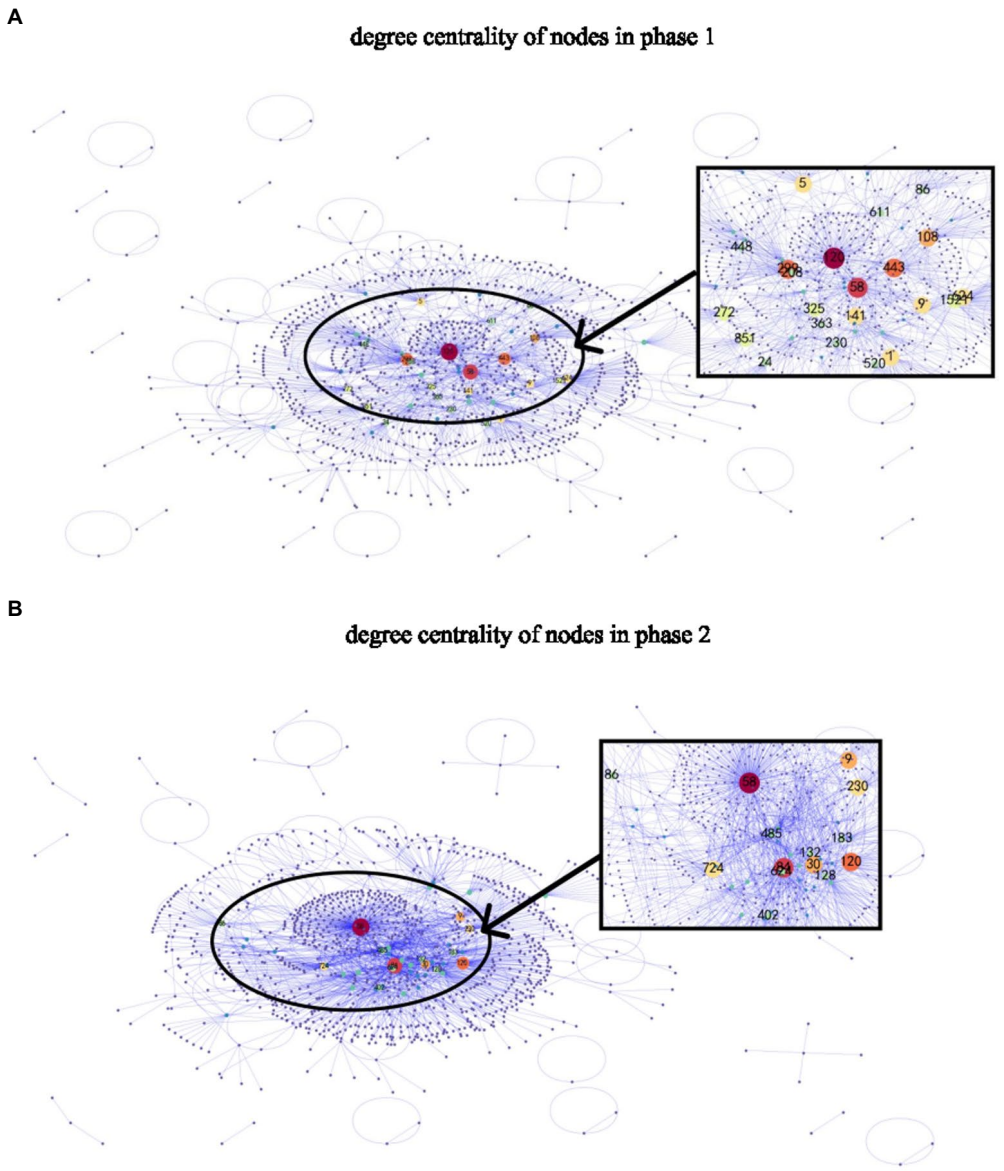
Degree centrality of nodes in **(A)** Phase 1, **(B)** Phase 2. The node size reflects degree centrality: the bigger the degree centrality value, the bigger the node size is. Users in both phases were randomly labeled. Note that nodes with big degree centrality in phase 1 remained robust and grew in size in phase 2, indicating increased influence and engagement of these users. 13 nodes remained to be hubs in both phases: 1, 9, 24, 30, 32, 58, 86, 120, 230, 383, 402, 624, 722.

The degree centrality means the number of edges connected to the nodes without considering the arrowhead. Nodes with a greater degree centrality remained robust and grew in size in phase 2 (see Figures 3A,B). Correlation results showed a significant correlation between in-degree and betweenness centrality (phase 1: *r*_219_ = 0.95, *p* < 0.001; phase 2: *r*_219_ = 0.99, *p* < 0.001); therefore, as betweenness increased, so would in-degree. Out-degree centrality and betweenness centrality did not consistently show significant correlation (phase 1: *r*_219_ = 0.40, *p* < 0.001; phase 2: *r*_219_ = 0.11, *p* = NS). Therefore, actors with higher in-degree centrality values showed a higher intermediary position in the network. Moreover, 13 hubs—the most connected nodes in each community (Montes et al., 2020)—in phase 1 remained in phase 2, indicating increased influence and engagement of these users. Hubs have important structural positions in each module, often associated with group control and stability functions (Fortunato, 2010).

**Figure 4:**
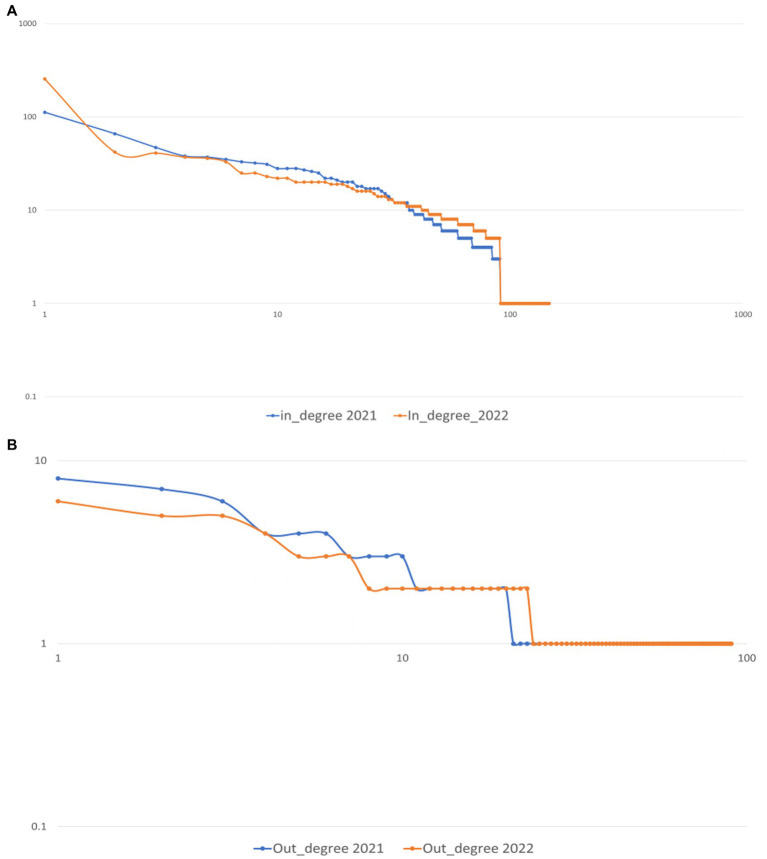
Log–log plot of the distribution of **(A)** in-degree of engaged users in both phases **(B)** out-degree of engaged users in both phases. Note in phase 2, there were fewer engaged users with low in-degree and out-degree. Compared to phase 1, there was a lack of a ‘long tail’ effect in the in-degree distribution in phase 2 **(A)**.

We present log–log plots for the in-degree and out-degree distribution of the engaged users (Figures 4A,B). Engaged users received more evenly distributed social support, and more users started to offer support to others: because in-degree is associated with receiving social support, and out-degree with offering support (Yang et al., 2017; Froehlich et al., 2020). Figure 4A shows fewer users with low in-degree in phase 2. Further evidence of more evenly distributed social support comes from the lack of a ‘long tail’ effect in the in-degree distribution in phase 2. Social support increasingly prevailed among the engaged users. In Figure 4B, the out-degree distribution shows a similar pattern, with more engaged users offering social support to others.

**Figure 5:**
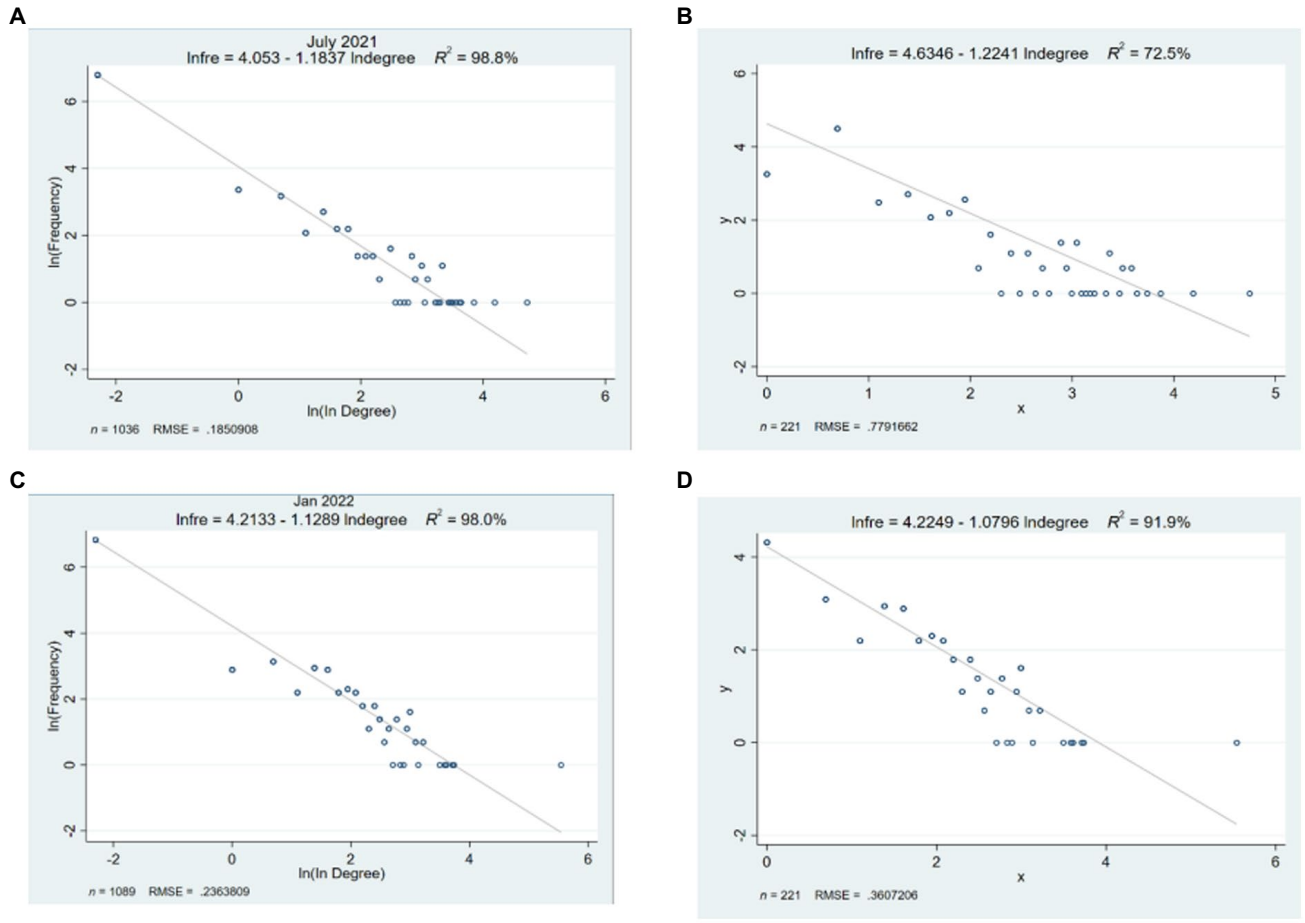
Power-law fit of cumulative degree distributions of **(A)** in-degree of the DSN (*N* = 1,036, phase 1); **(B)** in-degree of the subnetworks of the engaged users (*n* = 221, phase 1). Power law distribution of **(C)** in-degree of the whole DSN (*N* = 1,089, phase 2); **(D)** in-degree of the subnetworks of the engaged users (*n* = 221, phase 2). Power-law fit is shown as a plot of log degree (*x*-axis) by log cumulative degree distribution (*y*-axis). Left column = the DSN, right column = subnetworks of engaged users, top row = phase 1 networks, bottom row = phase 2 networks. Note for the DSN **(A)**, the slope for in-degree power law distribution was steeper in phase 1 compared to phase 2 **(C)**. For the subnetworks of engaged users, the slope for in-degree power law distribution was steeper in phase 1 **(B)** compared to phase 2 **(D)**.

Still, we have found an uneven distribution of social support in the DSN. There was a characteristic power-law ‘long tail’ in the DSN (see Figure 5). All log–log plots were close to linear. Edges were power-law distributed, resulting in few nodes having many edges and many nodes having few edges (Knoke and Yang, 2008). Louvain community detection provides further evidence of the uneven distribution of social support. Eighteen clusters in phase 1 and 16 clusters in phase 2 had less than three nodes (with one user and one commenter, or self-loops), indicating social support scarcity for some users in the DSN. However, the power-law fit suggested more evenly distributed social support for the DSN and subnetworks of engaged users. For the DSN, the slope for in-degree power law distribution was steeper in phase 1 (*α* = −1.18, *R^2^* = 0.99, see Figure 5A) compared to phase 2 (*α* = −1.13, *R^2^* = 0.98, see Figure 5C). For the subnetworks of engaged users, the slope for in-degree power law distribution was steeper in phase 1 (*α* = −1.22, *R^2^* = 0.73, see Figure 5B) compared to phase 2 (*α* = −1.08, *R^2^* = 0.92, see Figure 5D).

### 4.2. Results of content analysis

2. How would the changes in users’ self-disclosure reflect their mental well-being?

A descriptive analysis of self-disclosure attributes suggested a positive change in engaged users’ mental well-being (see Table 4). A decrease occurred in first-person singular forms, negative emotion words, and death words between phases 1 and 2. First-person singular forms can help detect depression since self-references are more frequent among depressed people: A person experiencing physical or emotional pain tends to focus more on themselves and thus use more first-person singular forms (Rosenquist et al., 2010; Leis et al., 2019; Tadesse et al., 2019). Less use of first-person singular suggests that users might experience less thus express less personal physical or emotional pain. There was a decrease in the use of death words meaning less expression of suicidal thoughts and attempts (Lumontod and Robinson, 2020). Emotions reflect the hedonistic aspect of well-being (Houben et al., 2015). Increasing positive emotion words and decreasing negative emotion words indicated positive change related to hedonic aspects of well-being. Words that reflect cognitive processing reveal the depth and complexity of people’s thinking, which reflects the eudaimonic aspects of well-being (Oliver, 2022). An increase in cognitive process words indicated positive changes related to well-being on the eudaimonic level.

**Table 4:** Descriptive analysis of self-disclosure attributes of the Depression SuperTalk Network in two phases and other social network sites.

Measures (LIWC code)	*M* (SD) phase 1	*M* (SD) phase 2		Twitter^B^	Expressive writing	Blogs
1st person singular pronouns (i)	5.19 (5.77)	4.35 (4.46)	↓	5.49	8.66	4.75
Death (death)	0.41 (1.41)	0.33 (1.58)	↓	0.15	0.12	0.19
Biological process (bio)	4.8 (5.77)	4.35 (4.46)		2.16	2.59	2.60
Positive emotion (posemo)	5.06 (6.75)	5.11 (8.89)	↑	3.66	2.57	5.48
Negative emotion (negemo)	6.33 (7.54)	5.09 (5.22)	↓	2.06	2.12	2.14
Cognitive process (cogproc)	18.54 (9.46)	19.49 (9.25)	↑	11.58	12.52	9.96
Adverb (adverb)	12.76 (8.35)	13.09 (8.06)		5.88	6.02	5.13
Informal (informal)	10.36 (8.61)	10.50 (8.06)		2.09	0.45	4.68
Active days	2.60 (3.48)	4.96 (6.00)		–^A^	–	–

A Data not available.

B The data related to Twitter, expressive writing and blogs are available in the LIWC-2015 manual, which draws on corpora containing million words as datasets, see Pennebaker et al. (2015) for details.

Previous research has found that using first-person singular pronouns, negative emotion words, and death words are important self-disclosure attributes that help reveal depression symptoms (Rosenquist et al., 2010; Leis et al., 2019; Tadesse et al., 2019). In this sense, engaged users showed less depression symptoms. There was a significant difference in the amount of negative emotion words between phase 1 (*M* = 6.33, *SD* = 7.54) and phase 2 (*M* = 5.09, *SD* = 5.22); *t*_220_ = 2.26, *p* = 0.03. There was a marginally significant difference in the amount of first-person singular forms between phase 1 (*M* = 5.19, *SD* = 5.77) and phase 2 (*M* = 4.35, *SD* = 4.46); *t*_220_ = 1.71, *p* = 0.08. The amount of death words also decreased from phase 1 (*M* = 0.41, *SD* = 1.40) to phase 2 (*M* = 0.33, *SD* = 1.58), but this difference did not reach statistical significance. Moreover, there was a significant difference in number of active days between phase 1 (*M* = 2.62, *SD* = 3.48) and phase 2 (*M* = 4.96, *SD* = 5.60); *t*_220_ = −12.82, *p* < 0.001, suggesting engaged users’ increased attachment and self-disclosure frequencies in the DSN.

The DSN was overwhelmed with death words, bio-words, and negative emotion words, compared to Twitter, blogs, and expressive writing (see Table 4). The overuse suggested that self-disclosure in the DSN featured topics related to depression, including negative emotions, body-related symptoms, and suicide narration. It was consistent with the group’s agenda to allow emotional venting, recovery, treatment, and social support among its members (Weibo Depression SuperTalk, 2022). The DSN also used more cognitive process words than other social network sites. One possible explanation is that the DSN is primarily a support group, unlike Twitter, blogs, or expressive writing. Cognitive process words were extensively used to provide suggestions, solicit social support, and self-reflection. We will conduct further research to investigate the differences in self-disclosure between the DSN and various social networking sites.

### 4.3. Combining SNA with content analysis

3. How can users obtain more social support through self-disclosure?

Overall, the results of SNA indicated that engaged users formed supportive relationships featuring improved connectivity, denser communities, non-centric and reciprocal interaction, and more frequent self-disclosure. Content analysis suggested that engaged users showed positive changes in mental well-being. It might be that these engaged users benefitted from online support when they engaged in the DSN in ways that fostered supportive interpersonal connections. To further access SNS’ effects on well-being, we run a correlation matrix and Poisson regression analysis with engaged users’ self-disclosure and topological attributes in phase 2, after they have stayed in the community for half a year.

**Figure 6:**
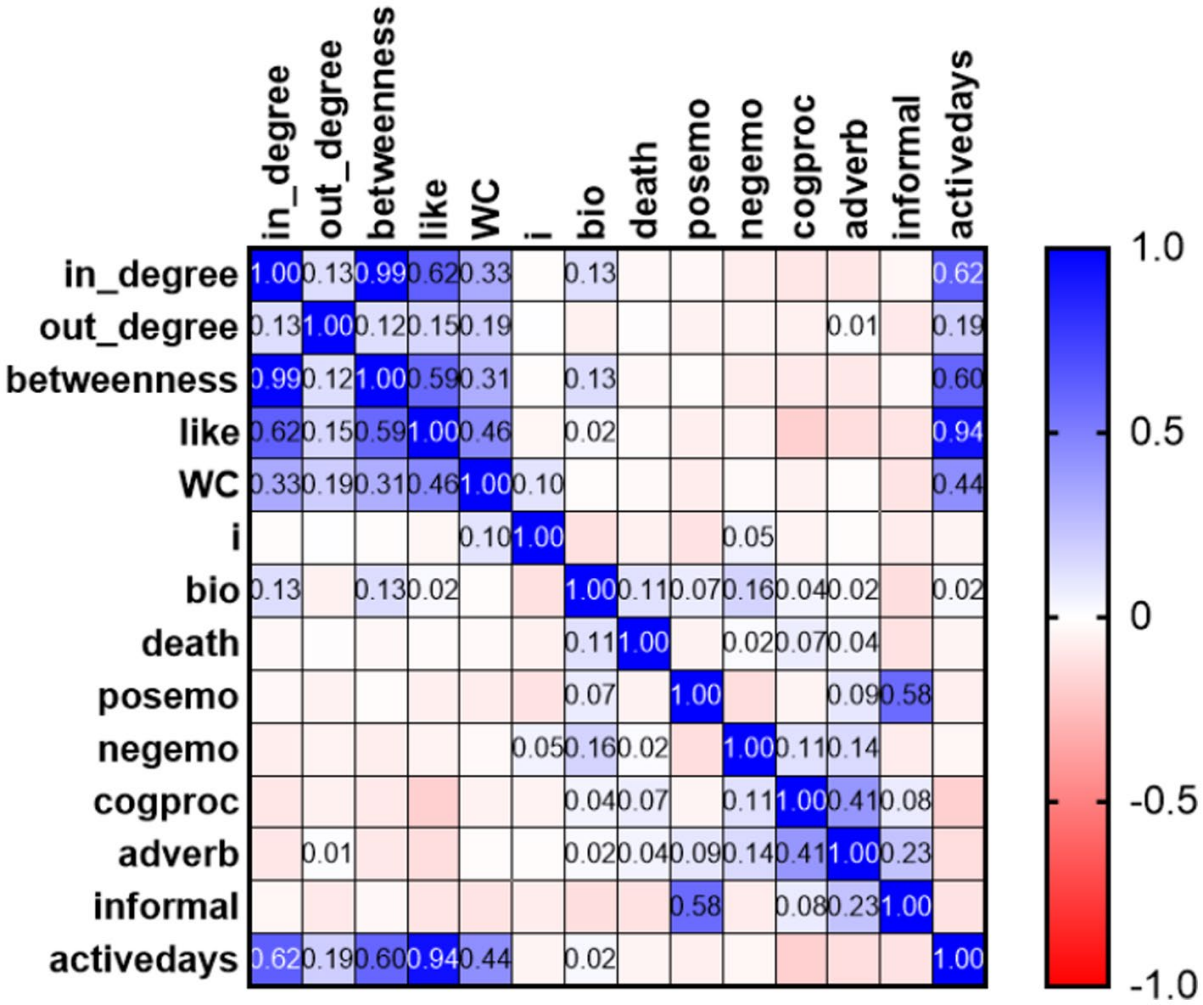
Correlation between users’ self-disclosure and topological attributes.

According to the correlation matrix, there were some correlations between topological attributes and self-disclosure attributes (see Figure 6). Significant correlations existed between the writing length and topological attributes, including in-degree (*r*_219_ = 0.33, *p* < 0.01), out-degree (*r*_219_ = 0.19, *p* < 0.05), betweenness-centrality (*r*_219_ = 0.31, *p* < 0.01), and likes (*r*_219_ = 0.46, *p* < 0.01). According to the correlation, users’ writing length appeared to impact their topological positions significantly.

Further evidence supporting the role of users’ content in their topological positions lies in strong correlation between the self-disclosure frequency (i.e., the number of active days) and topological attributes, including in-degree (*r*_219_ = 0.62, *p* < 0.01), out-degree (*r*_219_ = 0.19, *p* < 0.05), betweenness-centrality (*r*_219_ = 0.60, *p* < 0.01), and like (*r*_219_ = 0.94, *p* < 0.01). Based on the correlations, engaged users with a greater self-disclosure frequency were more likely to receive social support, provide social support to others, hold an intermediary position in the network, and receive emotional support from others.

Topological attributes and biological process words (bio-words) correlated, suggesting the homogeneity of the DSN. A significant correlation between bio-words and in-degree (*r*_219_ = 0.13, *p* = 0.01) indicated that users were more likely to receive social support when they posted health-related messages. The correlation between bio-words and like (*r*_219_ = 0.02, *p* < 0.05) suggested that sharing health-related information increased the likelihood of receiving favorable ratings and emotional support. The association between bio-words and betweenness-centrality (*r*_219_ = 0.13, *p* < 0.05) indicated that those who disclose health-related information would have a higher intermediary position and acted as a bridge for different clusters within the network.

After demonstrating how users’ self-disclosure affects their topological positions, the other side of the coin is to ask: how can users obtain more social support through self-disclosure? To answer the question, we ran a Poisson regression to predict users’ received social support based on their self-disclosure attributes. The Poisson regression method analyzes counts. Log of expected (mean) counts is modeled as a linear function of predictors, constraining predicted responses to be non-negative. Estimated coefficients represent the expected change in the log of the mean for a one-unit change in the corresponding predictor. Odds ratios (ORs) are estimated by exponentiating model coefficients in the inverse of the log link. Poisson regression results are expressed as rate ratios with 95% confidence intervals. Table 5 contains a summary of the Poisson regression results.

**Table 5:** Summary results of the Poisson regression.

Items	Coefficient	Std. Error	*z* value	*p*	OR	OR 95% CI
Ln (wc)	0.29	0.03	11.20	0.00	1.34	1.27–1.41
Informal	0.01	0.00	2.78	0.01	1.01	1.00–1.02
Adverb	−0.01	0.00	−2.64	0.01	0.99	0.98–1.00
Cogproc	0.00	0.00	0.15	0.88	1.00	0.99–1.01
Negemo	−0.03	0.01	−4.49	0.00	0.97	0.96–0.98
Posemo	−0.00	0.00	−0.04	0.97	1.00	0.99–1.01
Death	−0.01	0.02	−0.53	0.59	0.99	0.94–1.04
Bioproc	0.04	0.00	9.84	0.00	1.04	1.03–1.05
1st-person singular	0.03	0.01	4.05	0.00	1.03	1.01–1.04
Active day	0.09	0.00	22.34	0.00	1.10	1.09–1.11
Constant	0.28	0.13	2.24	0.02	1.33	1.04–1.70

Dependent Y: social support (in-degree). McFadden R2: 0.49.

In the final model, self-disclosure attributes associated with social support are: writing length (OR: 1.34; 95%CI: 1.27–1.41; *p* < 0.001), informal words (OR: 1.01; 95%CI: 1.00–1.02; *p* = 0.01), adverbs (OR: 0.99; 95%CI: 0.98–1.00; *p* = 0.01), negative emotion words (OR: 0.97; 95%CI: 0.96–0.98; *p* < 0.01), biological process words (OR: 1.04; 95%CI: 1.03–1.05; *p* < 0.01); first-person singular forms (OR: 1.03; 95%CI: 1.01–1.04; *p* < 0.01), and the number of active days (OR: 1.33; 95%CI: 1.04–1.70; *p* = 0.02).

The writing length is the strongest variable that influences users’ social support (*p* < 0.01; *β* = 0.29). It reflects users’ willingness to share information within the group, representing their desire for social support and emotional attachment to the community. The more information users self-disclose, the more engaged and interaction-motivated others will feel.

The number of active days is the second most significant variable affecting social support received by users (*p* < 0.01; *β* = 0.09). It is the number of days on which users post messages online. Those who more frequently show up and post are more likely to become acquainted, increasing their chances of receiving social support.

Biological process words (*p* < 0.01; *β* = 0.04) and first-person singular forms (*p* < 0.01; *β* = 0.03) also affect users’ social support. Users discuss depression-related symptoms, treatments, and embodied experiences. Health-related experiences fit the group’s common interests and would garner social support. People who express their concerns are likely to detail their experiences with depression in more depth, which will likely provoke others to recall similar experiences and obtain their social support.

Conversely, the use of negative emotion words (*p* < 0.01; *β* = −0.03) and adverbs (*p* = 0.01; *β* = −0.01) adversely impacts the social support received by users. The use of adverbs in expressions indicates extremeness; too much use may make others question the message’s validity. Adverbs have a negative correlation with like (*r*_219_ = −0.132, *p* < 0.05, see Figure 6), indicating lower odds of receiving favorable ratings when using more adverbs.

Lastly, informal words can also affect social support received by users. Using informal words makes the users appear courteous and well-mannered, which allows them to receive more social support online.

## 5. Discussion

Our study aims to assess SNS’ effects on well-being in light of the ongoing debate and ambiguous results (see section 2). The findings echo the need to bring in within-person analyses accounting for users’ personal and situational differences. SNS’ effects on well-being are not uniform or one-directional.

1. How would the dynamics of network structure strengthen or hinder social support?

With SNA, we have identified several network attributes strengthen social support, including network connectivity, global efficiency, degree centralization, hubs of communities, and reciprocal interactions. Network connectivity facilitates social support transmission with high global efficiency, a high average clustering coefficient, and a low degree centralization. The Louvain community detection revealed that the Depression Supertalk Network (DSN) formed stronger social ties and solid communities within communities (see Figure 2). Users clustered in more extensive and denser communities. Through social ties and interaction, smaller communities grew into larger ones. Reciprocity was also evidenced in the network, with significant correlations between in-reply and out-reply. When users post online, they are more likely to receive comments from others; and when users receive comments from others, they are more likely to respond. Log–log plots of in-and out-degree distributions (see Figure 4) showed that social support became more prevalent among engaged users: more engaged users received support and interacted with others. A less steep power-law slope of the in-degree distribution in phase 2 (see Figure 5) indicated an increasingly even distribution of social support.

Our findings are in accord with recent studies linking network attributes with social support on SNS. Individuals are surrounded by relationships that influence their health-related behaviors, social support resources, and health outcomes (Cobb et al., 2010; Phung et al., 2013; Froehlich et al., 2020; Li and Xu, 2020; Liu and Yeo, 2021). Several network attributes are found associated with strengthening social support, including network connectivity, degree centralization, global efficiency and reciprocal interaction (Lin et al., 2014; Li and Xu, 2020; Yang et al., 2021). Researchers have demonstrated that high network connectivity helps transmit valuable information among health professionals and allocate medical resources (Li and Xu, 2020). Several studies have examined how degree centralization and global efficiency affect users’ health behaviors and outcomes in online communities, illustrating the importance of non-centric and even support distribution. Meanwhile, reciprocal social support helps reduces loneliness and anxiety and improves well-being (Eysenbach et al., 2004; Malloch and Taylor, 2018; De Simoni et al., 2020).

Our finding suggests the strong impact of hubs on social support, which has not been sufficiently studied previously. Hubs, or the most connected nodes, significantly increased in degree-centrality and influence (see Figure 3). In each community, hubs were core users with prominent structural positions and played a crucial role in group control and stability. Up to now, several studies have begun to examine the importance of core users (Cobb et al., 2010; Carron-Arthur et al., 2015; Yang et al., 2017; Lin and Li, 2021). Gibbs et al. (2016) describe how certain core members are crucial to the development and sustainability of the online support community in a qualitative paper. Joglekar et al. (2018) identify users who are among the most connected in the online health support network. In the same vein, Levonian et al. (2021) find that long-term high-activity users are most vital to the sustainability of online health communities. Moving on to a better understanding of core users, Liang et al. (2019) propose to use a node-centrality analysis to identify core users and investigate the antecedents of their intention to stay active, from the perspective of social capital within enterprise-sponsored brand communities. A study by Lin and Li (2021) uses SNA and word co-occurrence network analysis to explore the characteristics of core users in tumor health communities. It finds that patients and their children were the most active groups. Based on a node-centrality analysis and the Louvain community detection method, the present study explores and proposes a different approach to identify core users. Future research is needed to investigate, using mixed designs, the mechanism of core users in online health communities, including their intentions to stay, longitudinal effects on group control and stability, and within-person differences.

The most disabling mental illness, depression is associated with low social support, especially when it has long-term adverse effects on close relationships (Kronmüller et al., 2011; Davidson et al., 2016; Lu et al., 2021). Consequently, our study contributes to the growing body of research suggesting the potential of online support groups for depression (Pan et al., 2020; Lu et al., 2021). Many easily accessible weak ties can provide support when people seek support in self-disclosure in a group-oriented environment, such as social network sites (Liu et al., 2021; Liu and Yeo, 2021). Friends and contacts in online support communities may indicate a higher level of social integration, allowing for more social support (Reifegerste et al., 2017; Hall and Liu, 2022; Parry et al., 2022). People with long-term conditions can benefit from joining online groups by being able to access social support. For those who lack or have limited access to offline support, the substitutability of offline illness work may be particularly helpful. A meta-synthesis of Allen et al. (2016) shows that social ties forged online enable individuals to engage in relevant self-management work, improve their illness experience, and address aspects of self-management that are difficult to meet offline. According to Prochnow et al. (2020), gamers who reported more site hours, more depression symptoms, and less offline support are significantly more likely to speak to other members about important life matters.

It should be noted, however, that we have also observed uneven distribution of social support among engaged users, although this was improving from phase 1 to phase 2. Several factors indicate an uneven distribution of social support, including a high number of communities with fewer than three nodes (Figure 3), a long tail effect in power-law distributions of in-degree and out-degree (Figure 4), and a power law fit of in-degree distributions (Figure 5). And in both phases, there was a lack of offering support to others as indicated by the lack of outbound links (i.e., out-degree) for red nodes (see Figure 1), as well as low out-degree (see Table 2). The number of users who offered social support increased, but the increase was offset by the number of target users to whom they provided support. Similarly, our results also revealed that out-degree had greater variability and range than in-degree (see Table 2). Users tend to be more homogeneous for out-degree (offer support to others) than in-degree (receive support from others). In other words, users were much less likely to offer support to others than receive others’ support in the community. We have found a big gap between receiving and offering support in the DSN, which confirms Hanneman and Riddle (2005) that SNS (e.g., Facebook, Twitter) tend to show more homogeneity regarding out-degree than in-degree. In the same vein, some studies have suggested that users would restrict online support to a limited number of users (Lin and Li, 2021; Lu et al., 2021; Ramamoorthy et al., 2021). The mechanism of low out-degree and its effects on SNS should be investigated in future research.

2. How would the changes in users’ self-disclosure reflect their mental well-being?

We have found within-person improvement in mental well-being manifested in self-disclosure in quality (valence, authenticity, intention) and quantity attributes (breadth, duration, frequency) (see Table 4). Among engaged users, a positive change in the hedonic aspect of well-being was manifested by more positive and less negative emotions among engaged users. Their increased use of cognition process words reflected a positive change in eudaimonic aspects of well-being. They used fewer first-person singular forms—a linguistic feature associated with depression. The decrease in death words showed less expression of suicide attempts and ideation among engaged users. Based on paired t-tests, there was a significant difference in the amount of first-person singular forms used and negative emotions expressed. A significant difference was also found between phase 1 and phase 2 in number of active days, indicating increased attachment to and self-disclosure frequencies in the DSN among engaged users.

In addition, we have compared self-disclosure in the DSN with that in other social networks, such as Twitter, blogs, and expressive writing (see Table 4). Overuse of biological process words and death words showed homogeneity among the group. It satisfied the group agenda of providing a forum for depressive users to share depressive experiences and discuss health-related issues, such as treatment, cure, and suggestions (Weibo Depression SuperTalk, 2022). SNS use has been shown to reduce the perceived riskiness of self-disclosure for people suffering from diseases stigmatized by culture and society, such as depression (Huang, 2016; Pan et al., 2017; Malloch and Taylor, 2018; Li et al., 2019; Chu et al., 2022). Online communities can also facilitate more appropriate and responsive support for individuals, since they can be formed around specific stressors rather than geographical locations (Liu et al., 2018; Lei et al., 2022). Posts, comments, and likes on SNS allow users to seek social support, respond promptly to requests from others, share emotional support, and provide tangible or intangible assistance to those in need.

Our findings bolster the call for understanding self-disclosure in a relational context to resolve the conflict over how self-disclosure affects well-being. For instance, although self-contained individuals have higher levels of depression and lower psychological well-being, authentic individuals have lower levels of depression and higher psychological well-being. Numerous studies have shown these relationships depend on whether their social networks are supportive or rejective. In relational contexts with partner responsiveness and engagement, self-disclosure leads to greater well-being, but not in those that lack virtual support (McConnell et al., 2018; Hou et al., 2020; Pechmann et al., 2020).

The effectiveness of SNS on well-being has been primarily demonstrated by shorter timeframe studies (e.g., days and weeks) (Prestin and Nabi, 2020). Researchers found that humans have a person-specific equilibrium point for well-being (Weinstein, 2018). The well-being of users drops when confronted with challenges (e.g., social media-induced stress) and rises when resources are available (e.g., online social support). For instance, it has been shown that within three-week, social media use can lead to both positive and negative effects on self-esteem. The effectiveness of SNS for social support and well-being is mixed in some longitudinal studies (van der Schuur et al., 2019; Beyens et al., 2020). As we examine the DSN over half a year, we suggest that situational within-person positive changes might occur through the long-term use of online support communities. However, with limited user size, caution must be applied. Once again, the findings corroborate with other research that indicates the extent to which SNS use is related to well-being depends on how individuals use it (Hall and Liu, 2022; Oliver, 2022; Parry et al., 2022; Valkenburg, 2022).

3. How can users obtain more social support to improve mental well-being?

Previous studies have primarily focused on the quantity of SNS use rather than users’ content or communication partners. Fewer studies combine users’ content and communication partners. Of the limited related research, Lin et al. (2014) suggest that users with larger networks on Facebook disclose more positive emotions and that a greater need for impression management explains the relationship between network size and emotional disclosure. Xu and Zhang (2016) find some correlation between the writing length and topological properties of group members. According to Pan et al. (2017), the bridging social capital of users (measured as network betweenness) is positively related to the responses they receive. In Pan et al. (2020), users’ informal language use affects the amount of social support they receive from their network. To provide richer language models and a more refined understanding of self-disclosure, we apply the Linguistic Inquiry and Word Count (LIWC-2015) to classify self-disclosure in qualitative and quantitative attributes. We have found several significant correlations between users’ self-disclosure and topological positions (see Figure 6). It appears that users’ self-disclosure plays a crucial role in establishing and maintaining relationships with others and forming social networks. Taking this further, we explore how users’ self-disclosure provides them with more social support, aiming for more practical implications (see Table 5).

The writing length was strongly correlated with one’s topological position, including in-degree, out-degree, and betweenness centrality. Similarly, the writing length largely affect one’s received social support. It lends credence to studies that consider message lengths a proxy for group integration and a measure of participation. Writing length reflects users’ intimacy and emotional connection to their DSN, which denotes social support (Lieberman, 2007; Batenburg and Das, 2015; Pan et al., 2017).

Biological process words correlated with betweenness (*r*_219_ = 0.13, *p* = 0.05): those who provided more health-related information would have a higher intermediary position and acted as a bridge between clusters. Meanwhile, biological process words significantly affect users’ received social support (*p* < 0.01; *β* = 0.04). This result occurred because homogeneity, rather than diversity, seems to be a particular attribute of the DSN. Users group together to share their common health concerns, which also fits the agenda of the DSN. Studies have shown that similar self-disclosure allows users to receive social support (Zhang et al., 2017; Malloch and Taylor, 2018; Zhang and Ahmed, 2018; Qu et al., 2022). Users are more likely to share their feelings, thoughts, and suggestions when reading a post that discloses similar health-related experiences. The perceived availability of support is more likely to increase if a person feels similar to and identifies with an online community. The similarity motivates people to seek out social connections and drives positive psychological outcomes (Malloch and Taylor, 2018; Liu et al., 2021).

The number of active days was also strongly correlated with topological attributes, making it a crucial factor in social support. Numbers of active days significantly differed between phases 1 and 2, indicating engaged users’ stronger attachment to the DSN. Posting more frequently online will likely transform weak relationships into stronger ones since users become acquainted more often. People are willing to offer support to members of an online community when they identify with its members (Pan et al., 2017; Pechmann et al., 2020). The benefits of active participation also appear to extend to users (Eysenbach et al., 2004; Carron-Arthur et al., 2015; Joglekar et al., 2018). In a study conducted by Chomutare et al. (2014), using social media tools for weight loss at least once a week is strongly associated with receiving encouragement. Marengo et al. (2021) find that active members of online support communities receive more responses than those who are less active. According to Lu et al. (2021), users who spend more time online and respond to other posts are more likely to form informational support ties.

Conversely, we have found that revealing negative emotions reduces users’ social support. There appears to be a consensus that expressing negative emotions will lead to more social support: human beings are particularly vulnerable to emotionally provoking content since negative emotions are open expressions of concerns and frustrations (Lieberman, 2007; Malloch and Taylor, 2018; Li et al., 2019). However, we are not alone in our view that expressing negative emotion might decrease users’ received social support. High et al. (2014) suggest that people are less likely to support individuals whose Facebook profiles disclose a greater range of emotions (versus fewer emotions). Lin et al. (2014) argue that Facebook users with larger networks on Facebook disclosed more positive emotions, and a stronger need for impression management mediates the relationship between network size and emotional disclosure. Ziegele and Reinecke (2017) find users are less willing to comment on negative status updates than on positive ones, moderated by the strength of the relationship between the sender and the receiver of the status update and mediated by perceived message appropriateness and support urgency. A recent study has found that if a person discloses too much negativity on social media, they may be perceived negatively and receive less support: people tend to present the positively valanced content on social media while concealing negative emotions (Pan et al., 2020). Once again, we have demonstrated the necessity of understanding self-disclosure in relational contexts. To better understand how self-disclosure of negativity affects social support in reconciling the aforementioned conflict, future studies are needed.

### 5.1. Implication

The theoretical implication of our study is the use of a relational approach to understanding the effects of social network sites (SNS) on well-being by accounting for users’ content (self-disclosure) and communication partners (social network). In our study design, we integrate data about the structure and composition of users’ online social support networks with users’ self-disclosure content (both in breadth and depth) to understand better the influence of online social support networks on health outcomes. A relational perspective on self-disclosure may provide researchers with a valuable framework for reconciling conflicting literature about the impact of self-disclosure. We demonstrate the significant role of social support in the link between self-disclosure and well-being, highlighting the importance of users’ self-disclosure features as they seek and receive social support. On the one hand, social network analysis (SNA), which takes account of users’ topological positions, uses network attributes to understand person-specific uses and group-based social support. We have identified users’ network attributes regarding providing and receiving social support in the context of depression. On the other hand, we have analyzed users’ change in mental health revealed in their self-disclosure, correlated their self-disclosure attributes with their topological positions, and anticipated how users’ self-disclosure attributes trigger others’ responsive social support. Self-disclosure facilitates the forming of relationships and maintains the dynamics of networks. Overall, we strengthen the need to analyze SNS’ effects on well-being with a relational approach: individuals’ well-being is intertwined with their relationships, surrounded by a network of relationships that influence their health outcomes and access to social support.

Our study also informs practices. As a starting point, users should pay attention to how they disclose since self-disclosure determines social support. Individuals’ topological positions are correlated with their content, suggesting they are surrounded by a network of relationships that impact their health outcomes. A comprehensive analysis of user networks and self-disclosure content indicates that digital connections are triggered by deliberate decisions to communicate instead of a chance encounter, like “bumping into someone serendipitously in a hallway— with no assumption that links are bidirectional” (Churchill and Halverson, 2005, pp. 18). The results of our study suggest that biological words, writing length, informal language, and active days indicate higher levels of social support; in contrast, negative emotions and adverbs indicate the opposite. These findings suggest that shared interests, intimacy, and reciprocal connection create a friendly semantic environment for online communities (Zhang et al., 2022). Due to the positive correlation between active days and out-degree, users can extend their ‘tenure’ in engagement with the community that serves their needs (Balani and De Choudhury, 2015). By referencing past self-disclosures and the community’s response to them, users can gain a sense of support when they visit online communities. Moreover, we have found a lack of offering social support versus receiving support in the group. To repay and exchange social support from communication partners, users can offer more social support to others. By doing so, individuals will have more trust and motivation toward the medium that mitigates self-disclosure and online support. Helping others can prevent feelings of helplessness and inadequacy and allow individuals to regain control and self-efficacy (Zhang et al., 2022).

Community organizers and web designers should cultivate intimacy and increase member reciprocity while encouraging users’ authentic self-disclosure. The sociograms created from our study are useful for visualizing connections within online health communities, which shows the persuasive power of mobilizing concepts such as social networks. In another case, Morris (2005) uses social networks to derive public displays of social interactions between elderly people and their relatives and friends. People see their social interactions illustrated in these feedback displays, and their feelings of social isolation are subtly and gently refuted. Moreover, active users can observe their topological positions in the network, realizing their contribution, potential, and connections with others, enhancing their mental well-being and community intimacy. The method used in this study can also help web designers and community managers predict fluctuations in growth and dropout among users and create a more pleasant, enjoyable, and integrated experience for users. Designers and web organizers can strengthen the bond between users and the community by recognizing and rewarding continuous participation (e.g., virtual currency). As part of specific social interactions, web designers and community organizers can also consider other self-disclosure context cues such as geographical location, temperament, gender, and hobbies. Users and communities might use these contextual cues in providing and receiving social support (Yoon et al., 2019; Pechmann et al., 2020). With multimodal self-disclosure features, including emojis, pictures, audio, and videos, users can better disclose themselves, receive social support, and engage in digital communication.

Also, this study provides valuable insight for public health surveillance and psychological treatment and support, exploring how online SNS interact with face-to-face support networks to influence health outcomes. We have identified several network attributes that strengthen social support, including network connectivity, global efficiency, degree centralization, hubs of communities, and reciprocal interactions. Based on the findings, public health professionals can devise targeted interventions. The relationship between users is particularly helpful for identifying influential people and determining the network’s closeness (Churchill and Halverson, 2005). The hubs of communities play an influential role in group control and stability. Placing them can facilitate the spread of accurate health information. Utilizing SNS data regarding health and well-being will enhance the future analysis of social support attributes. Psychotherapists can recognize the positive potential of online self-disclosure as a therapeutic ingredient and means of communicating with patients. With the client’s permission, psychotherapists may also use online self-disclosure texts to determine health-care interventions or as part of health outcome measurements.

Increasingly ingrained in digital life, our generation and the future have a major task: to adapt to digital life and utilize the good side of technology to flourish. Since the Internet enables people to engage with others across geographical boundaries, community members can draw upon the collective experience of participants who share a common health issue in a way that is impossible in the face-to-face world. This research has shown the potential of the online community for change: we have a group of engaged users who form intimacy and provide support to each other to cope with depression related anxiety, vent negative emotions and share valuable information. A strong correlation exists between depression and social isolation (Kronmüller et al., 2011; Davidson et al., 2016; Joglekar et al., 2018). SNS users, even those experiencing severe stress, repress their disclosures, negatively affecting their ability to obtain effective support (Calancie et al., 2017; Li et al., 2019). Engaged users in our study, by shaping their selective SNS use (deliberately or not), partly created their well-being effects through individual (i.e., self-disclosure attributes) and situational factors (i.e., communication partners). Online social support may have provided users with a sense of companionship and belonging, as well as reduced their long-term anxiety and enhanced their self-efficacy in coping with uncertainty in the future (Malloch and Taylor, 2018; Lu et al., 2021; Lei et al., 2022). The widespread impact of depression on individuals has led many to turn to social media for information, connection, and guidance, making online depression communities a promising new research area. In our study, we identify self-disclosure features that shape reciprocal, non-centric networks, which could also have implications for other telecommuting scenarios, including online education, entertainment, and working.

### 5.2. Limitations and future studies

This study has several limitations. Firstly, this study examines only one type of social network site. Future research might examine how various SNS designs may influence self-disclosure in its quality and quantity attributes. Community managers and platform designers can develop strategies for engaging group interaction and user participation by distinguishing differences in self-disclosure across various social networking sites. Further conceptual and methodological work can also study emojis, videos, and other multimodal disclosure features affecting SNS’s well-being.

Secondly, according to the official report, 80% of Weibo users are between 20 and 35, so our research might exclude adolescents and older users. Adolescents are more likely than adults to encounter negative experiences on SNS, such as cyber-victimization, sexing, and self-harm content (Beyens et al., 2020; Valkenburg et al., 2021). Adolescents’ omnipresence on social networks makes parenting increasingly challenging. Among older users who use SNS, executive function is positively predicted, but it also causes some older users to feel socially displaced (Hall and Liu, 2022; Oliver, 2022). It would be interesting to examine the use of social networking sites across different age groups and media-specific parenting and caregiving (van der Schuur et al., 2019).

Thirdly, we only consider limited personal (i.e., self-disclosure) and situational attributes (i.e., communication partners) when evaluating the impact of social networks on well-being, which might limit inferences to the general population. Although suggestive, our findings are correlational. It would be helpful to develop an integrated behavior change model that incorporates a variety of personal factors (ethnicity, diagnosis, treatment history, demographics, cognitive factors, and beliefs) as well as situational factors (e.g., culture, economy, professional, community, policy) (Lin et al., 2014; Li and Xu, 2020; Wang et al., 2021; Cingel et al., 2022; Hall and Liu, 2022; Parry et al., 2022). The future can use other modeling methods, such as dynamic structural equation modeling (DSEM), to demonstrate how many participants respond to an experimental design or to test certain hypotheses (Oliver, 2022; Parry et al., 2022; Valkenburg, 2022).

## 6. Conclusion

Social network sites have the potential to affect users’ well-being positively, but their effect is not uniform or one-directional. Our study combines individual (e.g., user self-disclosure) and situational (e.g., user social networks) factors to assess how SNS affects well-being. Three research questions guide the study and the paper. The first two show that self-disclosure is vital to establishing and maintaining relationships with others, as well as for the maintenance of networks. Our social network analysis reveals network attributes that improve social support; our content analysis reveals how self-disclosure, in its quality and quantity attributes, reflect users’ mental well-being. As part of the third research question, correlation analysis and Poisson regression analysis were used to determine how users’ self-disclosure attributes influence their topological positions and received social support. Users’ self-disclosure and network structure play a major role in enhancing social support for mental well-being, which is relevant to interventions and surveillance in public health. It is helpful for community managers to develop strategies for building communities, policymakers to disseminate health interventions, online users to seek social support, and researchers to study the situational effects of social support on well-being.

## Data availability statement

The original contributions presented in the study are included in the article/supplementary material, further inquiries can be directed to the corresponding author.

## Author contributions

JS: conceptualization, methodology, investigation, project administration, writing, review, and editing. ZK: conceptualization, methodology, formal analysis, and editing. All authors have read and agreed to the published version of the manuscript.

## Funding

This study was funded by The National Social Science Fund of China (Grant NO.: 20CYY016).

## Conflict of interest

The authors declare that the research was conducted in the absence of any commercial or financial relationships that could be construed as a potential conflict of interest.

## Publisher’s note

All claims expressed in this article are solely those of the authors and do not necessarily represent those of their affiliated organizations, or those of the publisher, the editors and the reviewers. Any product that may be evaluated in this article, or claim that may be made by its manufacturer, is not guaranteed or endorsed by the publisher.

## Footnotes

^1^Depression Supertalk (Weibo Depression SuperTalk, 2022), one of the largest health communities in Weibo, founded in Sep. 2019. According to its agenda, all members have self-tested or have been clinically diagnosed with depression. The community encourages its members to discuss problems, exchange suggestions, and share support to cope with depression.

^2^ https://anaconda.com

^3^ https://networkx.github.io

^4^ https://www.graphpad.com

^5^ https://www.ibm.com

^6^ https://www.butter.tools

^7^ https://www.liwc.app/
